# Cryo-EM structure of the ancient eukaryotic ribosome from the human parasite *Giardia lamblia*

**DOI:** 10.1093/nar/gkac046

**Published:** 2022-01-31

**Authors:** Disha-Gajanan Hiregange, Andre Rivalta, Tanaya Bose, Elinor Breiner-Goldstein, Sarit Samiya, Giuseppe Cimicata, Liudmila Kulakova, Ella Zimmerman, Anat Bashan, Osnat Herzberg, Ada Yonath

**Affiliations:** Department of Chemical and Structural Biology, Weizmann Institute of Science, Rehovot 7610001, Israel; Department of Chemical and Structural Biology, Weizmann Institute of Science, Rehovot 7610001, Israel; Department of Chemical and Structural Biology, Weizmann Institute of Science, Rehovot 7610001, Israel; Department of Chemical and Structural Biology, Weizmann Institute of Science, Rehovot 7610001, Israel; Department of Chemical and Structural Biology, Weizmann Institute of Science, Rehovot 7610001, Israel; Department of Chemical and Structural Biology, Weizmann Institute of Science, Rehovot 7610001, Israel; Institute for Bioscience and Biotechnology Research, University of Maryland, Rockville, MD 20742-4454, USA; Department of Chemical and Structural Biology, Weizmann Institute of Science, Rehovot 7610001, Israel; Department of Chemical and Structural Biology, Weizmann Institute of Science, Rehovot 7610001, Israel; Institute for Bioscience and Biotechnology Research, University of Maryland, Rockville, MD 20742-4454, USA; Department of Chemistry and Biochemistry, University of Maryland, College Park, MD 20742-4454, USA; Department of Chemical and Structural Biology, Weizmann Institute of Science, Rehovot 7610001, Israel

## Abstract

Giardiasis is a disease caused by the protist *Giardia lamblia*. As no human vaccines have been approved so far against it, and resistance to current drugs is spreading, new strategies for combating giardiasis need to be developed. The *G. lamblia* ribosome may provide a promising therapeutic target due to its distinct sequence differences from ribosomes of most eukaryotes and prokaryotes. Here, we report the cryo-electron microscopy structure of the *G. lamblia* (WB strain) ribosome determined at 2.75 Å resolution. The ribosomal RNA is the shortest known among eukaryotes, and lacks nearly all the eukaryote-specific ribosomal RNA expansion segments. In contrast, the ribosomal proteins are typically eukaryotic with some species-specific insertions/extensions. Most typical inter-subunit bridges are maintained except for one missing contact site. Unique structural features are located mainly at the ribosome’s periphery. These may be exploited as target sites for the design of new compounds that inhibit selectively the parasite’s ribosomal activity.

## INTRODUCTION

Ribosomes, the universal cellular protein synthesis molecular machines, are indispensable in all living cells. As ribosomes are crucial for cell survival, ∼40% of antibiotics in clinical use act mainly by binding to their functional centers (1). The distinction between bacterial and eukaryotic ribosomes has been used as the basis for antibiotic’s selectivity toward prokaryotic pathogens. However, general anti-ribosome drugs aimed at treating eukaryotic parasites lack selectivity owing to the high conservation of the targeted sites among eukaryotes. In addition, species specificity in the clinically used antibiotics is scarce, but specific properties (e.g. modes of antibiotic resistance) have been identified (2–4). Nonetheless, recent structural studies of ribosomes from bacterial pathogens have shown minor structural differences (among pathogens and compared with nonpathogenic bacteria), which could be leveraged for the design of novel species-specific antibacterial drugs (1,5–7). A similar approach, i.e. the targeting of unique structural differences among the eukaryotic pathogens and the human ribosome, can be used for the design of novel antiparasitic drugs.

*Giardia lamblia* (GL) is an anaerobic parasitic protist, which causes an acute human diarrheal disease called giardiasis and poses significant health and socioeconomic problems in poverty-stricken regions worldwide (8–10). The arsenal of drugs available for treating giardiasis includes only a few chemical classes, the 5-nitroimidazole, benzimidazole derivatives, quinacrine, furazolidone, paromomycin (PAR) and nitazoxanide. Quite often, these compounds have severe side effects, which leads to patient noncompliance, and in turn accelerates the spreading of resistance to these drugs (11–13). Only one of these approved drugs, PAR, targets the ribosome. However, treatment with PAR and other aminoglycoside derivatives induces side effects due to binding at ribosomal RNA (rRNA) small subunit (SSU) helix 44 (h44), which is highly conserved among prokaryotes, and eukaryotic and mitochondrial ribosomes as was previously shown in the structure of the eukaryotic parasite *Leishmania donovani* ribosome in complex with PAR (14). In contrast, several GL ribosome components have unique sequences, which may yield opportunities for discovery of selective anti-giardiasis compounds that are not subjected to the current GL drug resistance mechanisms. The high-resolution 3D structure of the GL ribosome facilitates the identification of novel ribosomal binding sites unique to this parasite that can guide this endeavor.

GL lacks mitochondria, and its genome encodes only cytosolic ribosomes, which are distinct from both eukaryotic and prokaryotic ones. The mitosome, a GL remnant of the mitochondria (15), does not include a translation machinery (16). Single-stranded RNA sequencing (17) and its nucleolar proteome analysis (18) indicate that GL is the most primitive eukaryote. Its rRNA is the shortest of any known eukaryote (i.e. compared to the human ribosome, GL rRNA is over 3000 nucleotides shorter), and is even shorter than that of all its bacterial counterparts (17). Moreover, the GL ribosome translation machinery differs from that of other known eukaryotic ribosomes, as it includes a reduced cohort of initiation factors and short UTRs, which dictate the structure of its mature mRNAs (19). The lack of some eIFs in GL is particularly striking because these translation machinery components are otherwise universally conserved and are essential in other eukaryotic life forms (20,21).

**Figure 1.**
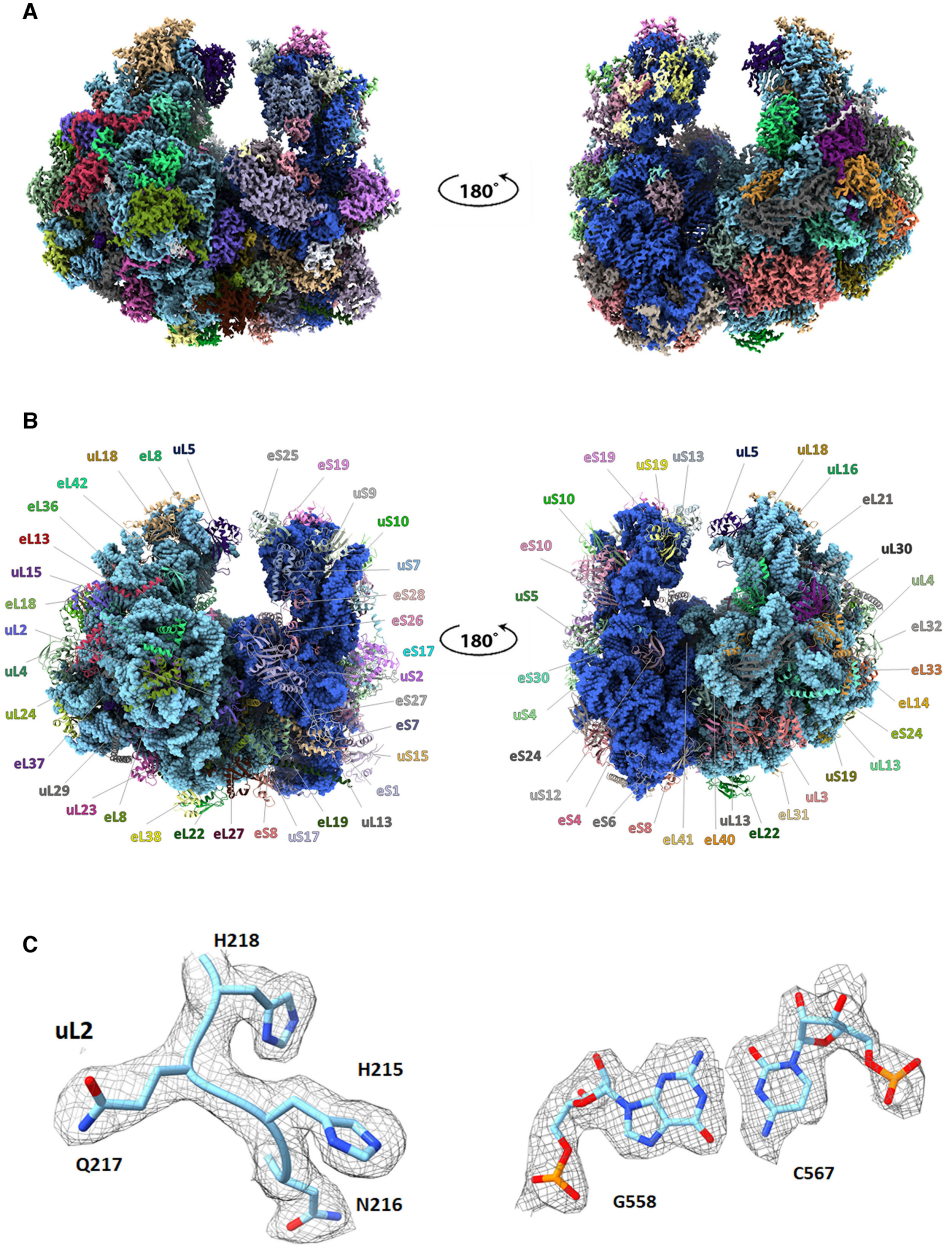
(**A**) Front and back views of the EM map of the GL ribosome colored according to the structure shown in panel (B). The map contour level is 2.2 *σ*. (**B**) Atomic structure of the GL ribosome, shown with the same front and back views as in panel (A). rRNA is shown as a space-filling model colored in blue, whereas ribosomal proteins (rProteins) are shown as non-blue colored ribbons. (**C**) Representative segments of the GL EM density map together with the associated atomic models of a protein tetrapeptide from the uL2 and a base pair of 28S rRNA. The map contour level is 2.2 *σ*.

This study reports the 2.75 Å resolution cryo-electron microscopy (cryo-EM) structure of the ribosome of the medically relevant GL isolate WB (Figure 1), which infects humans. As expected, we found that the GL ribosome comprises an extremely reduced version of typical eukaryotic ribosomes, missing nearly all of the eukaryote-specific rRNA expansion segments (ESs). Similar to rRNAs in other eukaryotes, some GL rRNA nucleotides are post-transcriptionally modified. Importantly, the positions of some of the modified nucleotides were identified directly in the EM density maps, as the identity of these modifications has not been previously known. These data provide structural insights for a better understanding of ribosome evolution across kingdoms. In addition, the structure offers a framework for the design of *Giardia* ribosome-targeting inhibitors.

## MATERIALS AND METHODS

### Purification of GL ribosomes

GL assembled A1 isolate WB trophozoites were cultured at 37°C using the previously reported protocol (22) except that the culture was expanded into cell culture five-layer flasks (Corning, Inc.) instead of borosilicate glass culture tubes, to enable production of sufficient amounts of ribosomes for the current study. Confluent culture was harvested by replacing the growth medium with lysis buffer A (45 mM HEPES, pH 7.6, 100 mM KCl, 10 mM MgCl_2_, 250 mM sucrose, 5 mM β-mercaptoethanol, 1:40 of RNasin solution), and chilling on ice for 20 min. Cell debris was removed by centrifugation and the cell lysate was layered on 1.1 M sucrose cushion buffer B (45 mM HEPES–KOH, pH 7.6, 100 mM KCl, 10 mM MgCl_2_ and 5 mM β-mercaptoethanol) and ultracentrifuged at 115 800 × *g* for 16.5 h in a Ti70 rotor at 4°C. The supernatant was discarded and the pellet was resuspended in buffer C [20 mM HEPES–KOH, pH 7.6, 150 mM KOAc, 10 mM Mg(OAc)_2_ and 5 mM β-mercaptoethanol]. Next, the resuspension was loaded on a 15%–50% sucrose gradient in buffer C and subjected to ultracentrifugation at 22 000 rpm for 11 h at 4°C on an SW28 rotor (Beckman). The 80S peak, which was shown to contain tRNA, although no intentional complex was preformed, was collected and centrifuged at 52 000 rpm for 16 h at 4°C. The pellet was resuspended in buffer D [20 mM HEPES–KOH, pH 7.6, 100 mM KOAc, 10 mM Mg(OAc)_2_, 10 mM NH_4_OAc and 1 mM DTT] and centrifuged for 1.5 h at 75 000 rpm on a TLA-100 rotor, in order to remove excess sucrose. The resulting ribosome-tRNA pellet was resuspended in buffer D and stored at −80°C at a final concentration of 3 mg/ml for further use.

### Cryo-EM data collection and refinement

3.5 μl of *Giardia* ribosome was added onto glow-discharged holey carbon grids (Quantifoil R2/2) coated with a continuous thin carbon film. Vitrobot Mark IV (Thermo Fischer Scientific) was used to blot and plunge freeze the grids. A Titan Krios electron microscope (Thermo Fischer Scientific) operating at 300 kV was used for collecting cryo-EM micrographs at liquid nitrogen temperature with a K3 direct electron detector (Gatan Inc.) at a nominal magnification of 105 000, with a pixel size of 0.85 Å/pixel and a dose rate of ∼1 electron/Å^2^/s. Defocus values ranged from −0.5 to −3.5 μm. Relion 3.1 (23,24) was used for data processing. Motion correction was performed using Motioncor2 (25). The contrast transfer function parameters were estimated using CTFFIND-3 (26). Semi-automatic particle picking followed by reference-free 2D classification resulted in a particle count of 260 691 that was used for building a 3D initial model (27). The particles were subjected to unsupervised 3D classification using a 60 Å low-pass filtered cryo-EM density map obtained from the initial 3D model as reference. Classes appearing to have well-formed 80S particles were selected. A total of 91 058 particles were used for auto-refinement. Following refinement, particles were subjected to CTF refinement, particle polishing and 3D refinement to calculate a 2.75 Å density map. The resulting 3D density map was then subjected to a cycle of multibody refinement (28) using separate masks for the large subunit (LSU) and the SSU, producing density maps at 2.7 and 2.9 Å resolutions, respectively. The map quality was improved, especially in the tRNA region. Also, the density of the pre-aligned 80S particles was subtracted, excluding tRNA binding pocket region. This was followed by second 3D classification, which allowed us to estimate the percentage of ribosomes that contained tRNAs. The gold standard Fourier shell correlation (FSC) value criterion of 0.143 was used for determining averaged map resolutions as implemented in Relion 3.1. Local resolution was estimated using Resmap (29).

### Model building and refinement

rRNA and rProtein structures were built by combining template-guided and *de novo* model building in COOT (30). The coordinates of the *Trichomonas vaginalis* ribosome [PDB codes 5XY3 and 5XY1 (31)] were used as a template for homology modeling with SWISS-MODEL (32) to obtain an initial model, which was then docked onto density maps using UCSF Chimera (33) and UCSF ChimeraX (34,35). EM density accounting for partial P- and E-tRNAs was identified in 99% of the particles, although no complex was preformed intentionally. Probably, harvesting the trophozoites during the log phase of growth yielded mostly actively translating ribosomes. The quality of the density map allowed only partial modeling of these tRNA molecules due to their high structural variability among the particles. RNA modifications were manually modeled. The Mg^2+^ and K^+^ ion compositions were modeled according to the recently described criteria (36,37). Model refinement was performed using an iterative approach, including real space refinement and geometry regularization in COOT, followed by real space refinement using the PHENIX suite (38). The final model was validated using MolProbity (39). The quality of the density map is depicted for a tetrapeptide and a base pair in Figure 1C.

## RESULTS AND DISCUSSION

### Overall structure of GL ribosome

To decipher the structure of the GL ribosome, intact ribosomes were purified and subjected to cryo-EM data collection. The initial data included 260 691 particles, yielding a 3.1 Å reconstruction of the entire ribosome, which was further refined and Bayesian polished to obtain an improved density map with an overall resolution of 2.75 Å for the entire particle (Supplementary Figure S1 and Table 1). Multibody refinement resulted in a slightly improved density map of 2.69 and 2.85 Å for the LSU and the SSU, respectively. The final density map and atomic models are shown in Figure 1A and B. Large regions of the density map, mainly located in the particle core, exhibited resolution better than 2.6 Å. A few highly flexible peripheral regions of the LSU showed resolution lower than 3.5 Å, and a few flexible peripheral regions of the SSU showed resolution lower than 4.5 Å (Supplementary Figure S2). The final density map accounts for ∼92% of rRNA components (18S, 5.8S, 5S and 28S rRNA), two partial tRNA molecules, and a total of 30 and 40 rProteins for the SSU and the LSU, respectively (Figure 1 and Supplementary Table S1). Inspection of the density map indicated that two tRNA molecules bound to the 99% of 80S particle (Supplementary Figure S3); however, while the E-site tRNA was more clearly visible, the density for the P-site tRNA was weak, implying either high flexibility or partial occupancy of the sites across the particle population (Supplementary Figure S3).

**Table 1.** Cryo-EM data collection and model refinement

Subunit	80S	LSU	SSU
**Data collection**			
Microscope		Titan Krios	
Camera		Gatan K3	
Voltage (kV)		300	
Magnification		105 000	
Pixel size (Å/pixel)		0.85	
Defocus range (μM)		−0.5 to −1.50	
Total dose (e/Å^2^)		45	
Micrographs collected		5567	
**Refinement**			
Number of particles (autopicked)		260 691	
Number of particles (used for 3D reconstruction)		91 058	
Resolution (Å; at FSC = 0.143)	2.75	2.69	2.85
CC (model to map fit)	0.79	0.87	0.79
**Model quality**			
Bonds (Å)	0.008	0.009	0.007
Angles (°)	1.090	0.970	0.943
Chiral volume outliers (°)	0	0	0
**Validation**			
Clashscore	8.00	3.77	6.92
**Proteins**			
MolProbity score	1.85	2.07	1.76
Rotamer outliers (%)	0.41	6.08	0.09
Ramachandran favored (%)	93.84	95.61	94.52
Ramachandran allowed (%)	6.06	4.37	5.30
Ramachandran outliers (%)	0.11	0.02	0.18
**RNA**			
Correct sugar pucker (%)	99.14	99.34	99.26
Correct backbone conformation	78.89	84.19	79.82

The structure analysis of the GL ribosome revealed an evolutionarily conserved architecture of the known universal functional sites, including the peptidyl transferase center (PTC) and the decoding center, which are formed by the rRNA (40). In this respect, most clinically used antibiotics that bind to these conserved functional sites are expected to bind similarly to the *Giardia* and to the human ribosomes, and thus lack drug selectivity. Superposition of the GL decoding center on the human and the *L. donovani* counterparts shows the structural similarity of the PAR binding site (Supplementary Figure S4). Note that the *L. donovani* invariant nucleotides A2158 and A2159 are flipped out of the rRNA helix due to the PAR binding. Such rearrangement is a common feature of aminoglycosides that bind the decoding site (14,41–44) and we expect similar nucleotide flipping to also occur upon PAR binding to the GL ribosome.

### The GL compact rRNA

Analysis of the SSU rRNA has led to the proposal that GL resembles the common ancestor of prokaryotes and eukaryotes (45), which is considered by some as the missing link between them (46). Although this view has been contradicted and refined over the years (47), GL is still considered by many to represent early branching eukaryotes that diverged from a common ancestor before the acquisition–retention of mitochondria (46). The elucidated GL ribosome structure exhibits unique features that add new perspective into the evolutionary dilemma. By superpositioning of their rRNA structures, we show that the GL rRNA is shorter than the typical prokaryotic (i.e. *Escherichia coli*), the typical parasitic (i.e. *L. donovani*) and the fungal rRNA (i.e. *Saccharomyces cerevisiae*) (Supplementary Figure S7). While working on the current manuscript, an additional structural study of the GL ribosome, at lower resolution, was published by Eiler et al. in bioRxiv, which discussed a lower amount of adenines and uridines compared to other species and concluded that all the important eIF3 binding sites are conserved (48).

**Figure 2.**
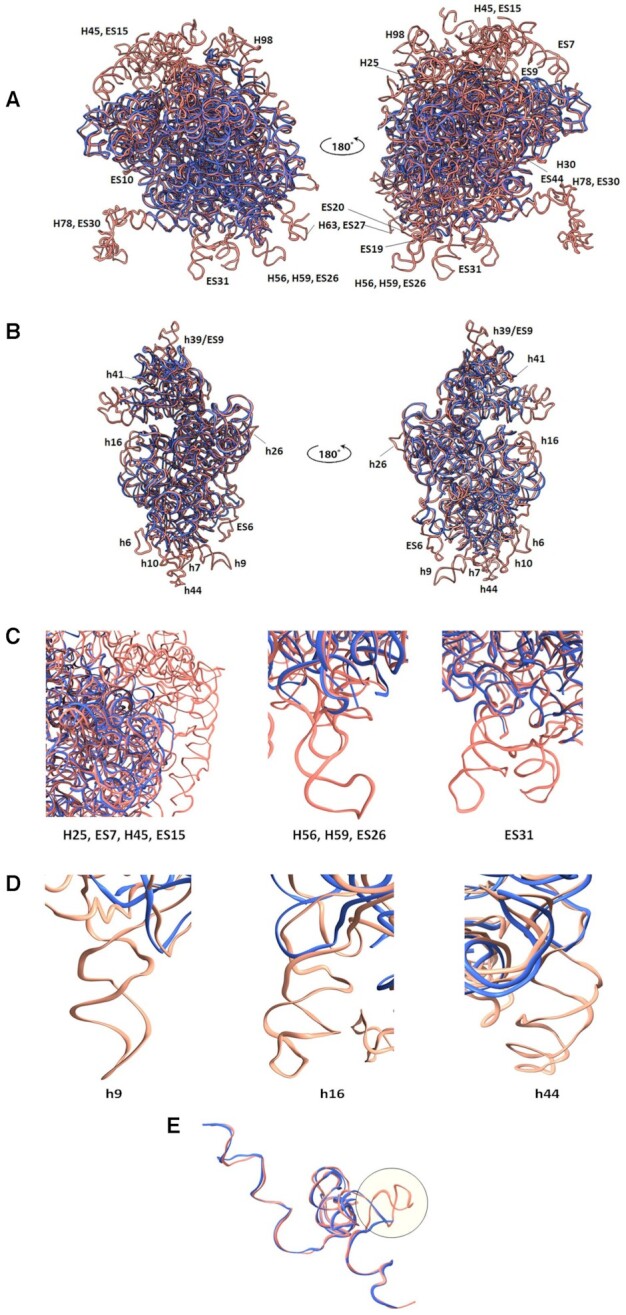
Large differences between the GL and the human ribosomal rRNA (PDB ID 4UG0) structures are located mainly at the particle’s periphery. (**A**) Superposition of the backbones of the GL (blue) and the human (salmon) LSU and (**B**) SSU ribosome structures. The absent GL rRNA helices and ESs are labeled. (**C**) Close-up view of the missing H25, ES7, H45, ES15, H56, H59, ES26 and ES31 in GL (blue) and human (salmon) 28S rRNA. (**D**) Close-up view of the missing h9, h16 and h44 in GL (blue) and human (salmon) 18S rRNA. (**E**) 5.8S rRNA of GL (blue) is shorter than the human counterpart (salmon).

Since the comparison between the GL rRNA and human rRNA structures is most relevant for future drug development efforts, we focus on their specific structural differences. Thus, the comparison shows that many rRNA ESs present in typical eukaryotic SSUs, which are located within the foot and head regions in the orientations shown in Figure 2, are absent in the GL ribosome (Figure 2A–C). Furthermore, the GL LSU has almost no rRNA ESs at the solvent-exposed surface of the subunit (Figure 2B–D) and the 5.8S rRNA structure is truncated compared with the human 5.8S rRNA (Figure 2E); however, the base pairing with 28S rRNA is conserved. In addition to the striking differences of the eukaryote-specific ESs, the GL ribosome includes shorter helices and even lacks many of them compared with the human ribosome. In the SSU, rRNA helices h6, h16, h17, h40, h41 and h44 are shorter than those of the human counterparts, whereas helices h9, h10, h21 and h39 are absent (Figure 2B). In the LSU, helices H14, H25, H30, H49, H63, H98 and H99 are shorter and exhibit different structures compared with the human counterparts, whereas helices H59 and H78 are missing (Figure 2A).

### The GL divergent rProteins

Although GL appears to branch from other eukaryotic classes early in evolution, all of its rProteins include globular domains that are conserved in eukaryotes. Albeit some of the proteins have variable N-terminal or C-terminal extensions, most are structurally identical to their human counterparts (a few examples are shown in Supplementary Figure S5). However, two eukaryotic rProteins are absent, i.e. eL6 and eL28, as discussed later. In addition, whenever specific eukaryotic ESs are missing, the rProteins that stabilize these regions show unique structural variance if compared to their human counterparts.

**Figure 3.**
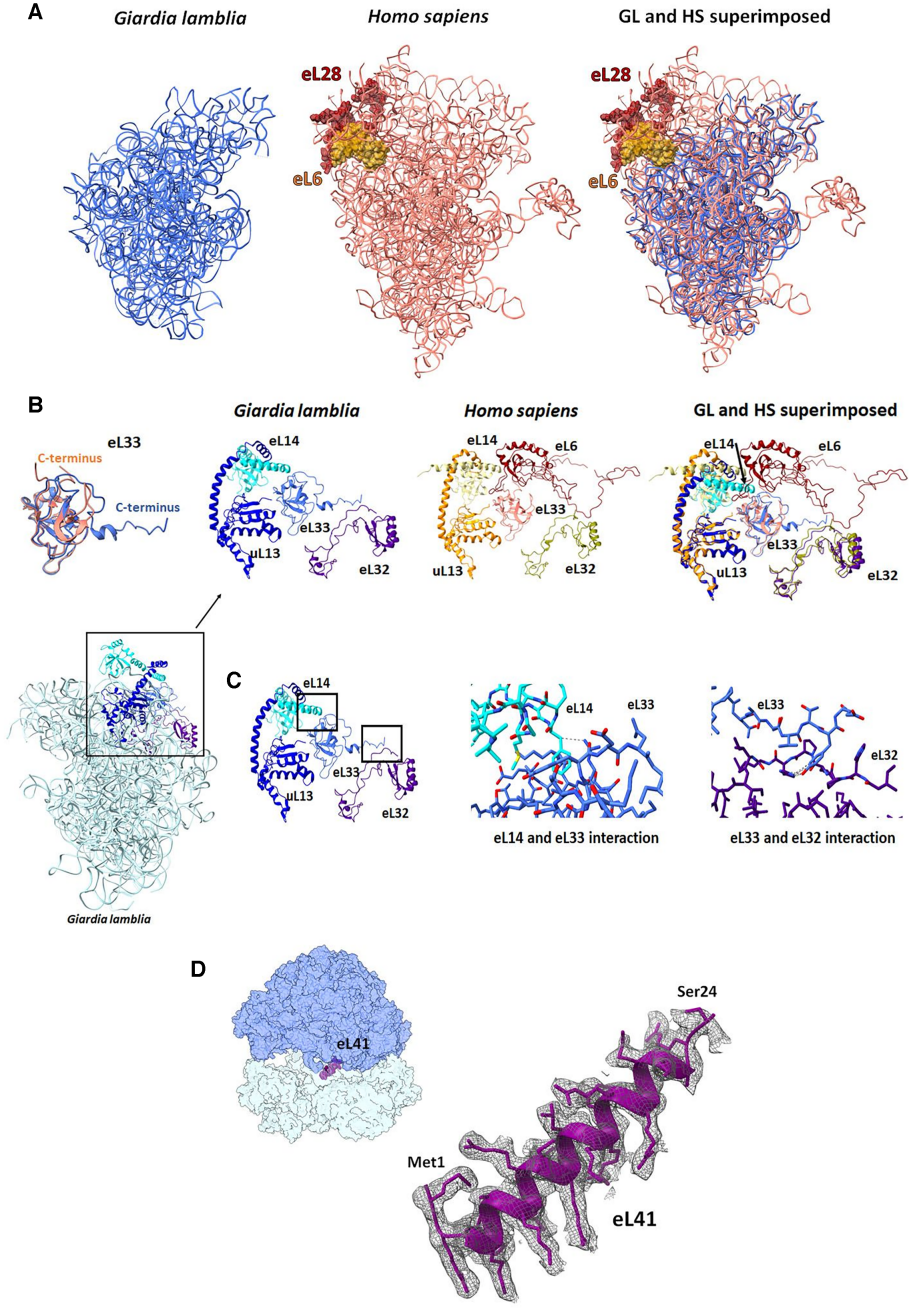
(**A**) eL6, eL28 and some ESs in the human ribosome (salmon) (PDB ID 4UG0) are missing in the GL ribosome (blue). (**B**) GL eL33 (blue) C-terminus adopts a different conformation compared with the human eL33 (salmon). The eL6, present in the human ribosome, is absent in GL. (**C**) The zoom-in images (marked with black squares) show the detailed specific interactions within the GL ribosome between eL33, eL14 and eL32. Possible interactions between eL14, eL33 and eL32. (**D**) EM density and structure of the hypothetical protein assigned as eL41. The map contour level is 2.2 *σ*.

Most notably, the human rProteins eL6 and eL28, which are absent in the GL ribosome, bind and stabilize the extruded region of ES7–ES15 of the human ribosome that is located on the solvated face of the LSU. Because of the missing rProteins and the associated rRNA ESs, the GL ribosome protein–protein and protein–RNA interactions in that region diverge dramatically from those in the human ribosome (Figure 3A).

The GL ribosome contains an extended rProtein eL33 C-terminus compared with the human eL33 (Figure 3B and Supplementary Figure S6A). In the absence of rProtein eL6, this terminal region interacts directly with the C-terminus of eL32. Moreover, rProtein eL14 C-terminus also shifts to interact with rProtein eL33 (Figure 3C). Conversely, in the human LSU, rProteins uL13 and eL14 interact through their C-termini, and rProtein eL14 makes no contact with rProtein eL33 due to the presence of a loop of rProtein eL6. Instead, rProtein eL6 interacts with rProteins eL33 and eL32, whereas eL32 and eL33 do not interact directly.

Notably an elongated density located at the inter-subunits fits very well with the structure of eL41 from the human ribosome (Figure 3D). Because of its small size, the GL protein was annotated in the GL protein database as a longer hypothetical protein (gene ID: GSB_151147). The cryo-EM structure shows that this protein is shorter than the annotated one and its start codon for translation may be annotated as eL41, which is part of the B3 and eB14 inter-subunit bridges (Supplementary Table S2).

**Figure 4.**
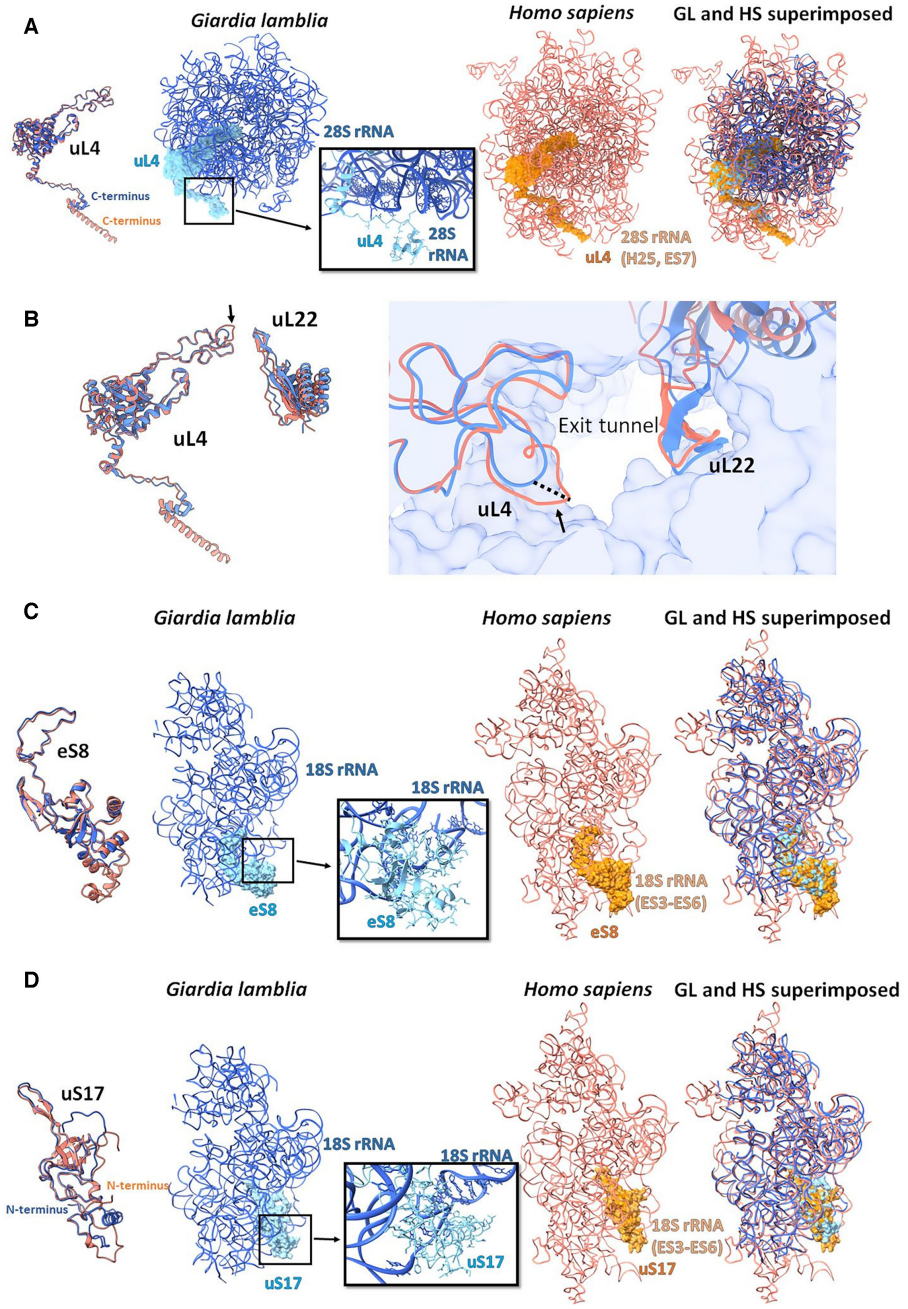
Large differences between the GL (blue) and the human rProteins (salmon) (PDB ID 4UG0). Compared to the human counterparts, (**A**) GL uL4 has a shorter C-terminus, (**B**) GL uL4 has a shorter loop at the exit tunnel, (**C**) GL eS8 has a shorter loop and (**D**) GL uS17 has an extended N-terminus. The zoom-in boxes show the detailed specific interactions within the GL ribosome using the ball and stick presentation.

The uL4 C-terminus exhibits a striking difference between the GL and human rProteins. In GL, the C-terminus wraps around the solvent-exposed rRNA region at helix H25. In contrast, the human uL4 C-terminus adopts an extended helical conformation and is embedded within rRNA regions that are entirely missing in the GL ribosome (Figure 4A and Supplementary Figure S6B). Additionally, a uL4 loop that protrudes toward the LSU exit tunnel is shorter in GL compared with the human uL4 loop. Consequently, the GL ribosome’s exit tunnel is wider than that of the human ribosome and hence is an excellent target site for developing *Giardia*-specific drugs (Figure 4B).

Furthermore, the GL SSU lacks the human ES3–ES6 and its eS8 protein is truncated compared with the human counterpart, where eS8 interacts with the ES3–ES6, which are thought to be late evolutionary extension loops (49) (Figure 4C and Supplementary Figure S6C). Conversely, the GL SSU rProtein uS17 N-terminus is extended compared with the human uS17, perhaps because the human uS17 interacts with ES6, which is missing in the GL SSU (Figure 4D and Supplementary Figure S6D).

Taken together, from the evolutionary aspect, the GL ribosome appears to be a ‘compact’ or a minimal eukaryotic ribosome. The rRNA comprises shortened 18S, 5.8S and 28S rRNA, whereas the set of eukaryotic proteins is almost but not entirely complete. In fact, the rRNA is even shorter than the typical prokaryotic rRNA (Supplementary Figures S7 and S8), supporting the hypothesis that GL represents an example of earlier evolutionary branching organisms from a common ancestor compared with high eukaryotes and even most parasitic eukaryotes.

### Inter-subunit bridges in the GL 80S ribosome

**Figure 5.**
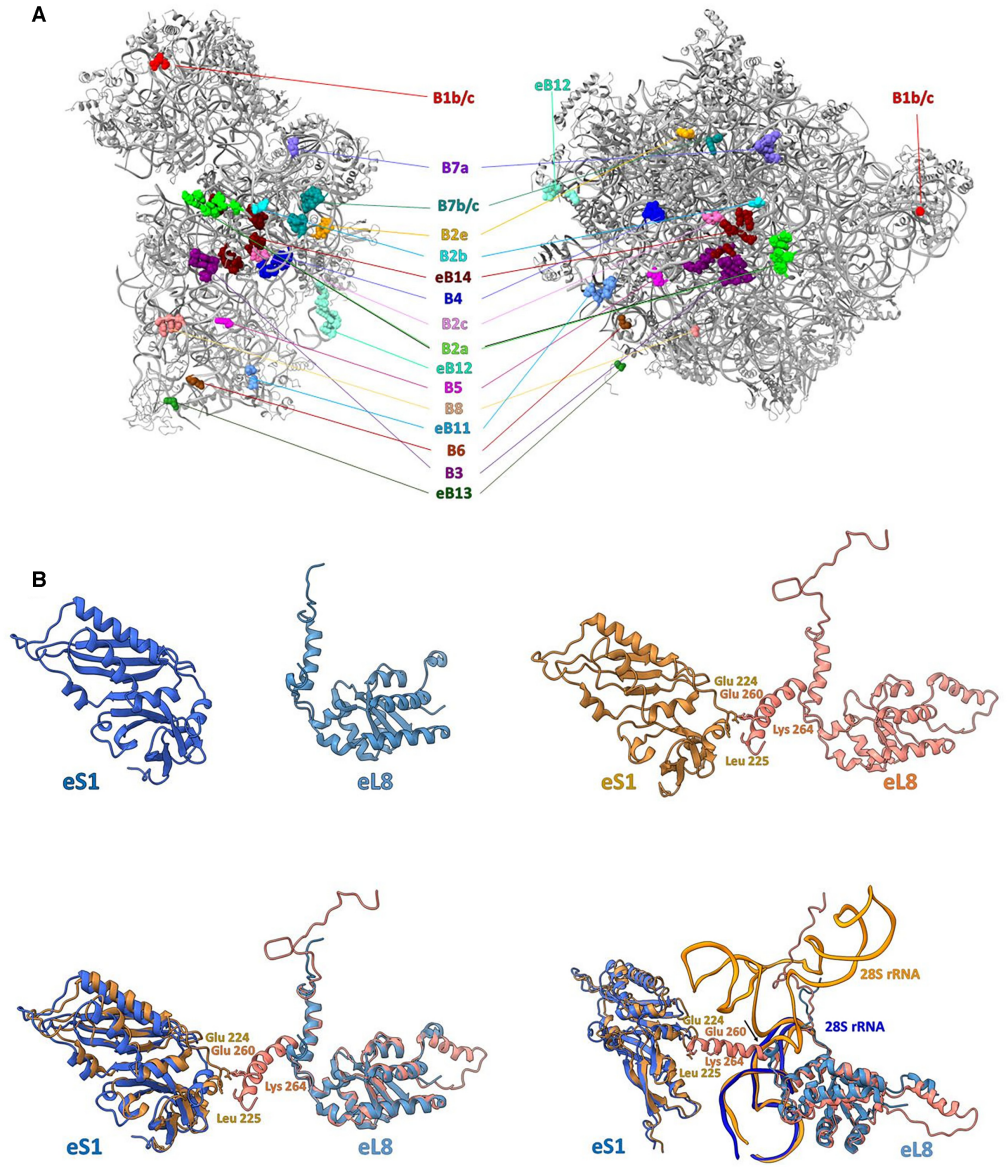
(**A**) Inter-subunit bridges in the GL ribosome. The atoms of each bridge, shown as space-filling models, are colored using identical colors for both the SSU and LSU and are labeled accordingly. (**B**) Eukaryote-specific eB8 bridge (right, red and pink) (PDB ID 4UG0) and missing in the GL ribosome (left, blue and light blue). The superposition of the rProteins that mediate the eB8 bridge is shown at the bottom left. The proposed region that can be used as a target for drugs is pointed with the arrow and is shown at bottom right.

Inter-subunit contacts between SSU and LSU play a major role in forming a functional ribosome, in conveying conformational signals during the protein synthesis and in regulating various aspects of translation (50). We compared the inter-subunit bridges in GL to those in known structures of eukaryotic and prokaryotic ribosomes and found that most of the previously reported bridges (50–54) are well preserved in the GL ribosome (Figure 5A and Supplementary Table S2) despite the lack of many eukaryote-specific rRNA ESs. Importantly, many RNA–RNA, protein–RNA or protein–protein bridges, mainly at the peripheral region, display substantial differences, which could be exploited as potential sites for drug development (Supplementary Figure S9). However, the only case of a protein–protein inter-subunit bridge that is missing in the GL ribosome is that of eB8, involved in two contact points between eL8 and eS1 in the human ribosome (52). These contacts are absent in the GL ribosome; therefore, we propose that this region may be targeted by compounds that would not bind to the human ribosome (Figure 5B). Thus, targeting of the rRNA of this peripheral large subunit region may selectively interfere with GL subunit interactions and inhibit GL ribosome activity in a similar fashion to what has been suggested for inhibiting the bacterial ribosome (55).

### rRNA modifications in GL 80S ribosome

**Figure 6.**
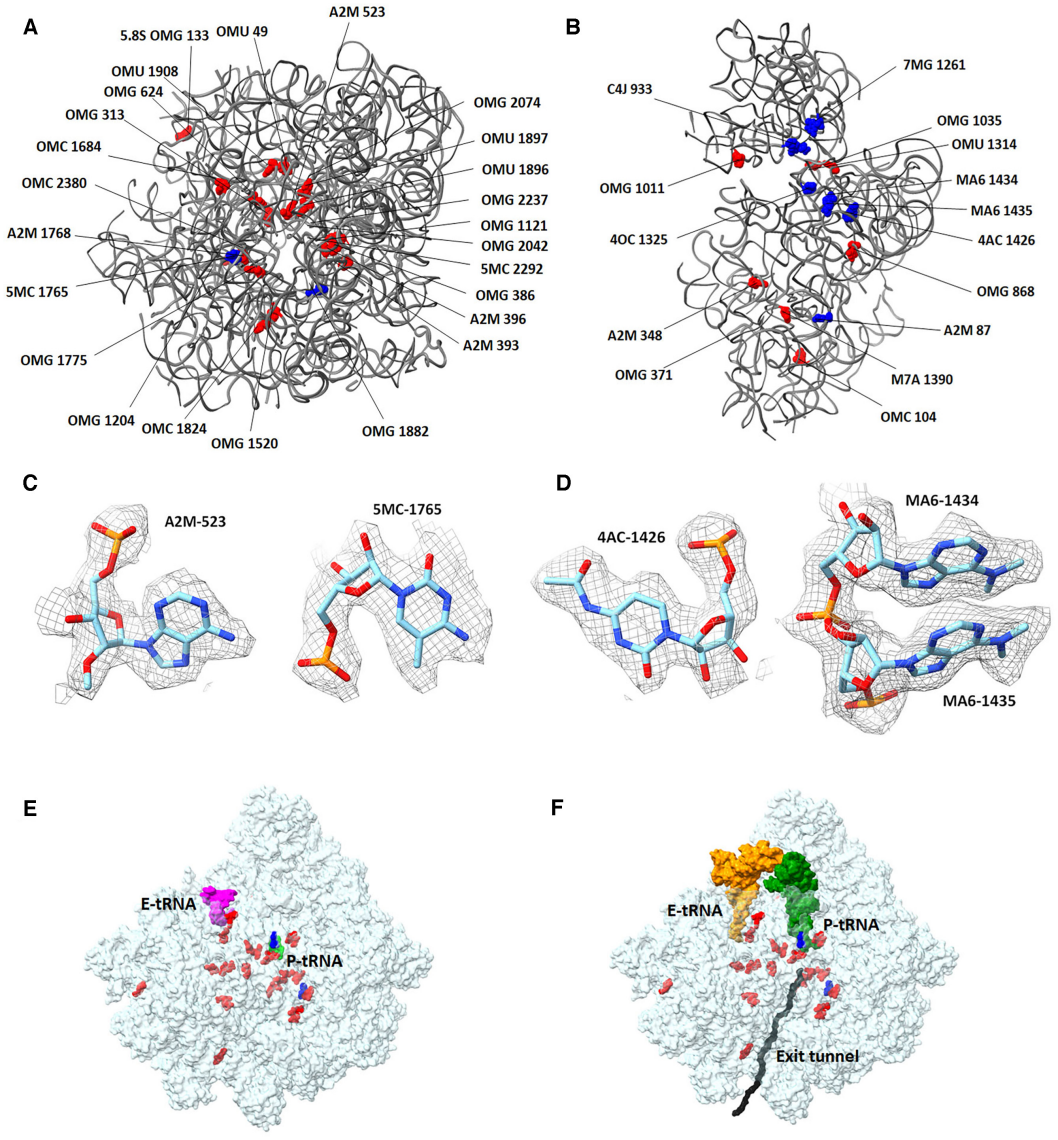
rRNA modifications modeled in the GL ribosome structure: (**A**) LSU and (**B**) SSU rRNA. The sugar modifications are shown in red, and base modifications are shown in blue. (**C**) LSU examples of modified rRNA, i.e. A2M-523 and 5MC-1765 as modeled in GL ribosome EM density map. The map contour level is 2.2 *σ*. (**D**) SSU examples of rRNA modifications modeled within the EM density map, i.e. 4AC-1426, MA6-1434 and MA6-1435. The map contour level is 2.2 *σ*. (**E**, **F**) LSU rRNA modifications have been modeled mostly within the PTC and around the protein exit tunnel. (**E**) The P- and E-site tRNAs were modeled based on partially interpretable density map seen in 99% of the GL ribosome particles. (**F**) The P- and E-site tRNAs were *in silico* superposed from PDB ID 6ZJ3 and shown for orientation.

Studies of bacteria and archaea have shown that modified rRNA plays an essential role in the catalytic activity and ribosome biogenesis (56–60). Structural studies of *E. coli* (61) and *T. thermophilus* (62) ribosomes have shown the importance of rRNA modifications in bacterial ribosome integrity and function. Most rRNA modifications in the bacterial ribosomes are localized at or near functional sites, including the PTC, the peptide exit tunnel and the mRNA–tRNA interaction site that are involved in the decoding center (61,62). These studies also verified the effect of rRNA modifications on the conformations of the pockets where various antibacterial agents bind. In general, the number of rRNA modified nucleotides within the prokaryotic ribosome is very small compared with eukaryotic ribosomes, where modifications on sugar groups as well as pseudouridines are rather common (63,64). However, some eukaryotic organisms, with rRNA composed of several segments, contain exceptionally high number of rRNA modifications [e.g. the *L. donovani* (65) and *Euglena gracilis* (66) ribosomes], which were proposed to assist the proper ribosome biogenesis and the stabilization of the fragmented rRNA ribosome particles by additional RNA–RNA and RNA–rProtein interactions. Although rRNA modifications are widespread across species, their positions and chemical composition are less conserved. Prior to our studies, no experimental data about the GL rRNA modification pattern were available. However, using modification patterns of the *L. donovani* and the human ribosomes as references enabled modeling of a significant number of modified rRNA nucleotides in the GL ribosome EM density map (Table 2). Most GL rRNA modifications, although not all of them, are localized in and around functional regions in the interior of the ribosome rather than on its periphery (Figure 6). In case of differences with the human rRNA modification, the structures will assist with the design of selective GL ribosome inhibitors.

**Table 2.** RNA modifications in *Giardia* ribosome

rRNA	Modified nucleotide number	Nucleotide	Type
28S rRNA	49	Um	OMU
	313	Gm	OMG
	386	Gm	OMG
	393	Am	A2M
	396	Am	A2M
	523	Am	A2M
	624	Gm	OMG
	1121	Gm	OMG
	1204	Gm	OMG
	1520	Gm	OMG
	1684	Cm	OMC
	1765	Cm	5MC
	1768	Am	A2M
	1775	Gm	OMG
	1824	Cm	OMC
	1882	Gm	OMG
	1896	Um	OMU
	1897	Um	OMU
	1908	Um	OMU
	2042	Gm	OMG
	2074	Gm	OMG
	2237	Gm	OMG
	2292	Cm	5MC
	2380	Cm	OMC
5.8S rRNA	133	Gm	OMG
18S rRNA	87	Am	A2M
	104	Cm	OMC
	348	Am	A2M
	371	Gm	OMG
	868	Gm	OMG
	933	Um	C4J
	1011	Gm	OMG
	1035	Gm	OMG
	1261	Gm	7MG
	1314	Um	OMU
	1325	Cm	4OC
	1390	Am	M7A
	1426	Cm	4AC
	1434	Am	MA6
	1435	Am	MA6

OMG, *O*2′-methylguanosine 5′-monophosphate; OMU, *O*2′-methyluridine 5′-monophosphate; OMC, *O*2′-methylycytidine 5′-monophosphate; A2M, 2′-*O*-methyladenosine 5′-(dihydrogen phosphate); 5MC, 5-methylcytidine 5′-monophosphate; C4J, (5*S*)-5-{3-[(3*S*)-3-amino-3-carboxypropyl]-1-methyl-2,4-dioxo-1,2,3,4-tetrahydropyrimidin-5-yl}-2,5-anhydro-1-*O*-phosphono-l-arabinitol; 7MG, 7*N*-methyl-8-hydroguanosine 5′-monophosphate; 4OC, 4*N*,*O*2′-methylcytidine 5′-monophosphate; 4AC, *N*^4^-acetylcytidine 5′-monophosphate; M7A, 7-methyl, adenosine 5′-monophosphate; MA6, *N*^6^-dimethyladenosine 5′-monophosphate.

## CONCLUSION

Ribosome sequences, specifically the SSU rRNA, have long been used as key markers for assessing evolutionary processes. The GL ribosome structure provides the first 3D glimpse into a group of ribosomes from organisms at a branching point that separates prokaryotes and eukaryotes. Strikingly, the rProteins and the rRNA show contrasting trends as most of the proteins are very close to proteins of high eukaryotes, including humans, whereas the rRNA is much less elaborated and is even shorter than the prokaryotic rRNA chains. From a practical perspective, the GL ribosome structure along with the available human ribosome structures provides a framework for a structure-based discovery of drugs that specifically inhibit the activity of the GL ribosome and cure giardiasis. The new structure revealed several potential binding sites, including within the exit tunnel and the different inter-subunit bridges, which so far have not been exploited by the pharmaceutical industry, and thus should open new avenues for developing *Giardia*-specific therapeutic agents free of side effects, which are also not subject to current GL drug resistance mechanisms.

## DATA AVAILABILITY

The cryo-EM density maps of the GL ribosome have been deposited in the Electron Microscopy Data Bank (EMDB) under accession numbers EMD-13683, EMD-13681 and EMD-13680. Atomic coordinates and structure factors have been deposited in the Protein Data Bank (PDB) under accession codes 7PWO, 7PWG and 7PWF.

## Supplementary Material

gkac046_Supplemental_Files
