# Supplementary material for: Cryo-EM structure of the ancient eukaryotic ribosome from the human parasite Giardia lamblia
Source: Nucleic Acids Res. 2022 Jan 31;50(3):1770–82. doi: 10.1093/nar/gkac046 (PMC8860606; doi:10.1093/nar/gkac046)
Supplement: gkac046_Supplemental_Files [file gkac046_supplemental_files.zip › 100122_GL_Supp.pdf]

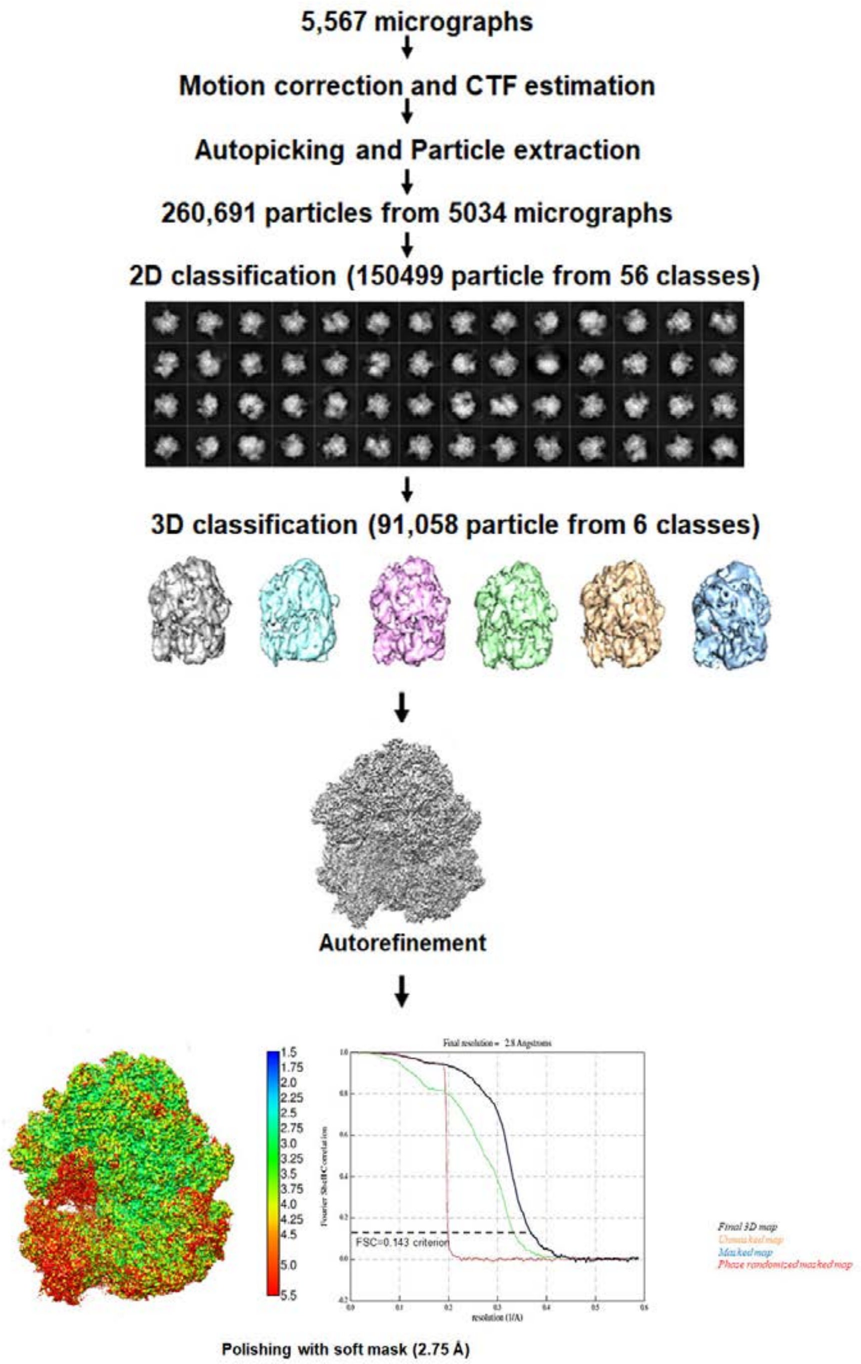

Supplementary Figure S1. *The cryo-EM image reconstruction flow chart for the GL ribosome.*

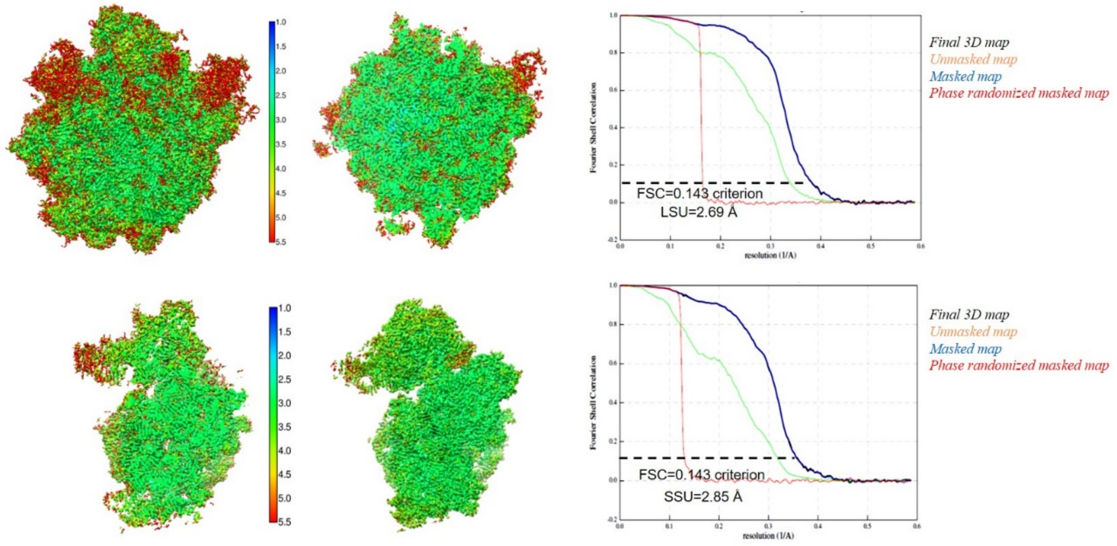

Supplementary Figure S2. *Local map resolution and Fourier Shell Correlation (FSC) curves. Surface (left) and cross-section (right) rendering of the cryo-EM density maps of GL colored according to local resolution distribution. "Gold standard" FSC curves for the final 3D map (black), unmasked map (green), masked map (blue), and phase randomized masked map (red) indicate nominal resolutions of 2.69 Å and 2.85 Å (FSC=0.143 criterion) of the masked map. The map contour level is 2.20 σ.*

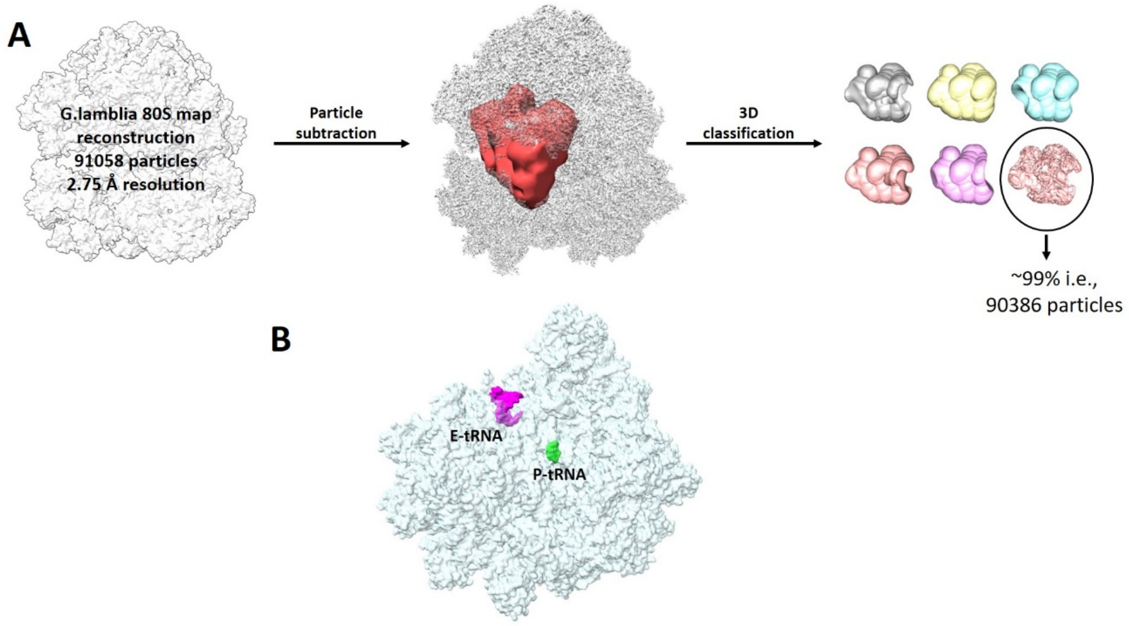

Supplementary Figure S3. *(A) Focused refinement for the tRNA binding pocket to estimate the percentage of occupancy of tRNA in the 80S map. (B) E and P-tRNA partial structures as modelled within the large subunit of the GL ribosome EM density map.*

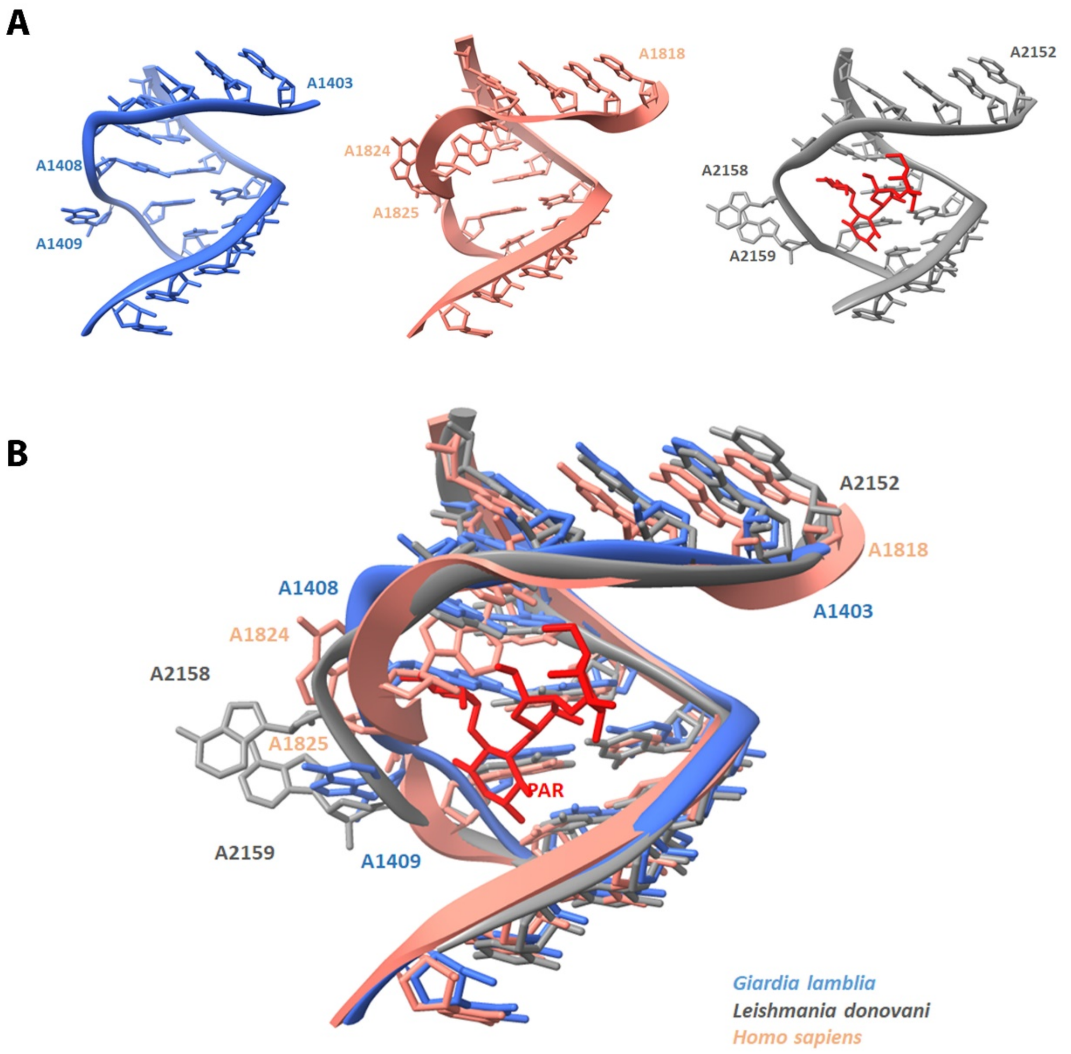

Supplementary Figure S4. *Superposition of the L. donovani in complex with PAR (PDB ID 6AZ1) shown in red on the GL and human (PDB ID 4UG0) decoding centers, of which the structures closely resemble one another. (A) The individual structures, (B) superposition of the 3 structure shown in (A).*

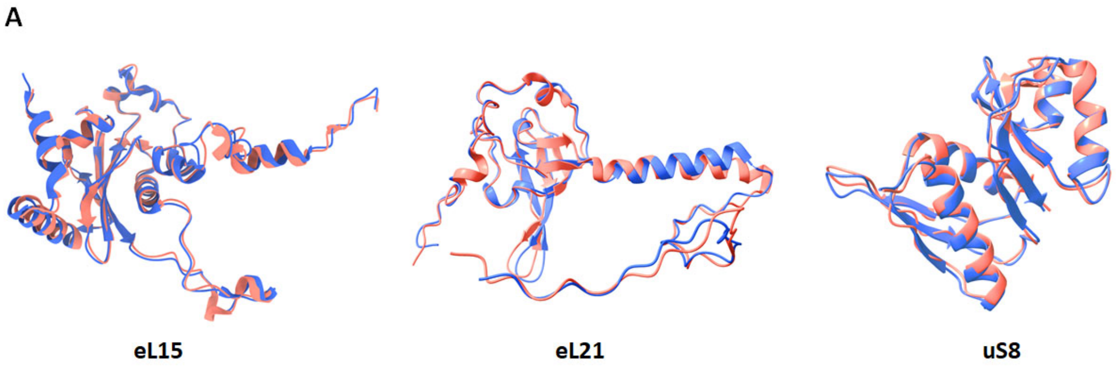

Supplementary Figure. S5. *examples of GL rProteins that are structurally similar to their human (PDB ID 4UG0) counterparts.*

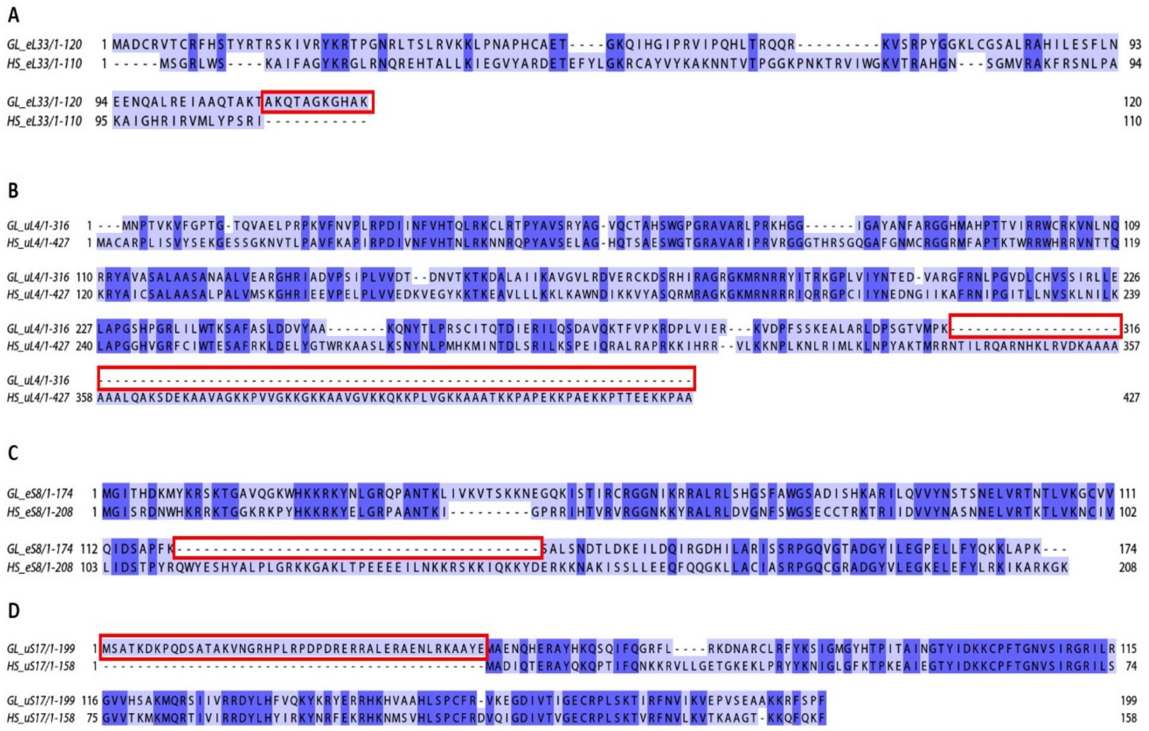

Supplementary Figure S6. *Pairwise alignments of divergent rProtein in the GL ribosome and their human counterparts. (A) GL eL33 has an extended C-terminus. (B) GL uL4 has a shorter C-terminus. (C) GL eS8 has a missing loop. (D) GL uS17 has an extended N-terminus.*

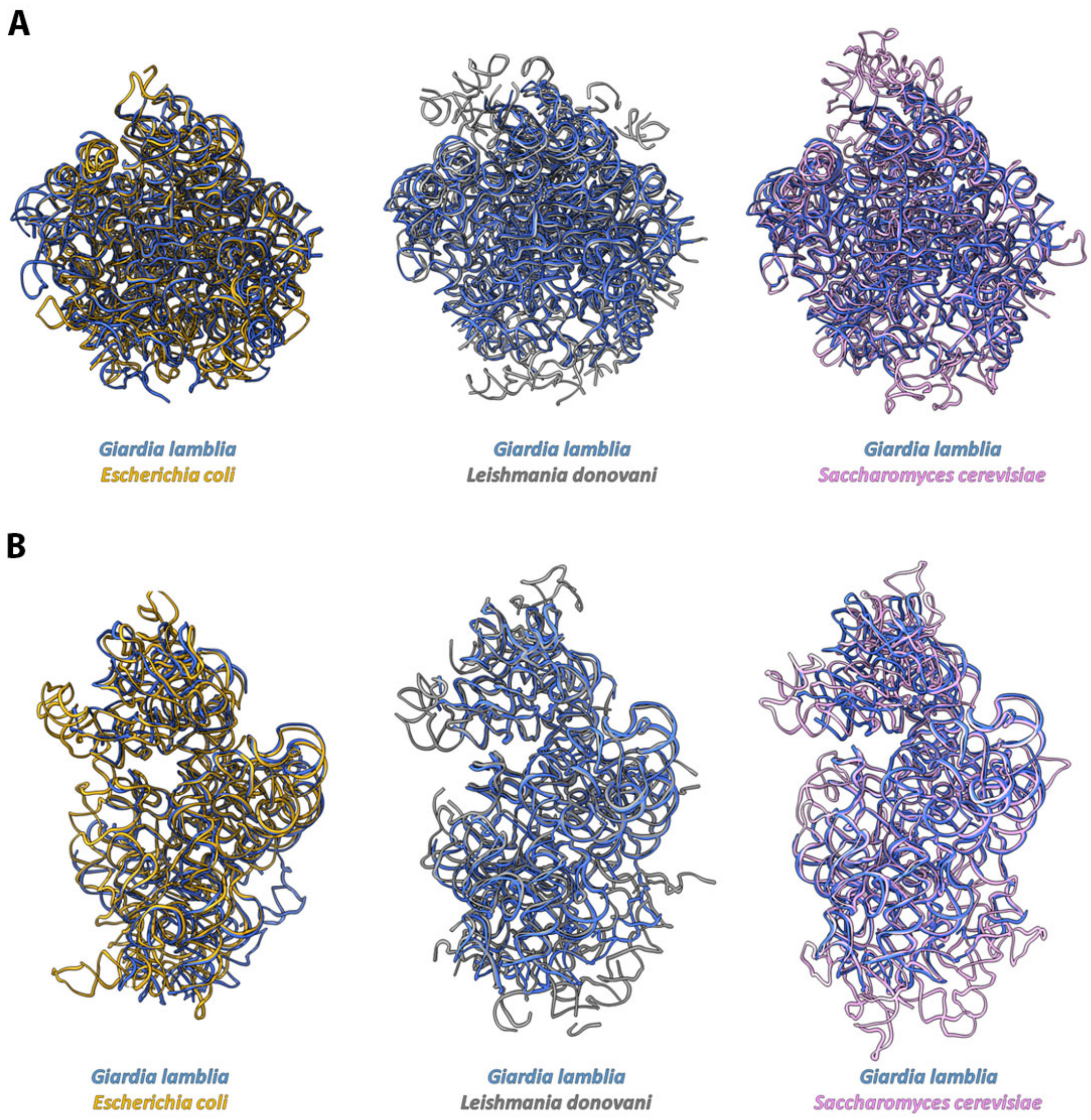

Supplementary Figure S*7. Comparison of GL rRNA (blue) with E. coli rRNA (PDB ID 7k00, Golden), L. donovani (PDB ID 6AZ3 (LSU) and 6AZ1 (SSU), grey) and S. cerevisiae (PDB ID 4V88, pink). (A) and (B) are the superpositions of LSU and SSU, respectively.*

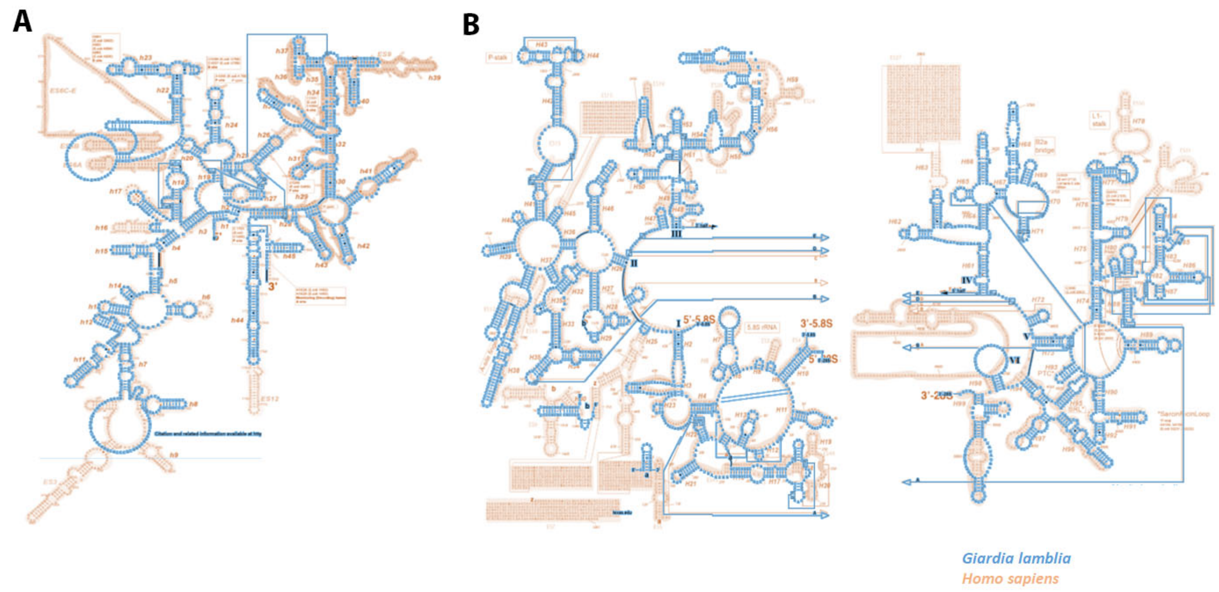

Supplementary Figure S8. *Comparison of 2D map of GL rRNA (blue) with Homo sapiens (pale orange). (A) and (B) are the superposition of LSU and SSU, respectively.*

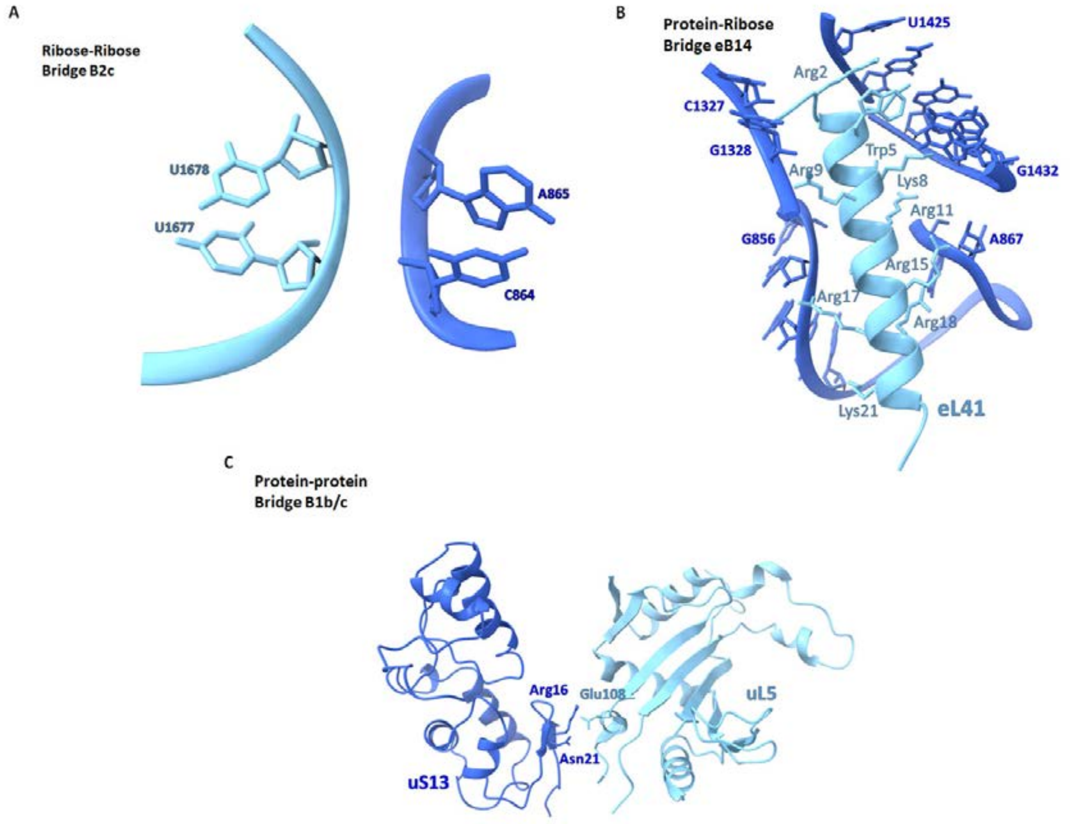

Supplementary Figure S9. *Molecular details of inter-subunit contacts found in GL ribosome. (A) Bridge B2c highlighting rRNA:rRNA between 28S rRNA (Light blue) and 18S rRNA (dark blue). (B) Characteristic protein–RNA interaction between 18S rRNA region and protein eL41 in bridge eB14. (C) Protein:protein bridge B1b/c, which involves proteins uS13 and uL5.*

Supplementary Table 2: Inter-subunit bridges specific interactions in the *GL* 80S ribosome*.

Bridge	40S subunit		60S subunit	
**B1a**	not observed			
**B1b/c**	uS13 (RPS18)	Arg 16, Asn 21	uL5 (RPL11)	Glu 108
**B2a**	18S	1410-1412, 1433-1434, 1329-1331	28S	1749-1750, 1742, 1747
**B2b**	18S	746	28S	1682
**B2c**	18S	865-866	28S	1677-1678
**B2e**	18S	633-634	eL43	Arg 85
**B3**	18S	1399-1400, 1341-1343	28S	1788-1790, 1777-1779, 1612-1615
			eL41	Arg 23
**B4**	18S	510-511, 723-724	28S	562-563
**B5**	18S	1353	28S	1566
**B6**	18S	1377	eL24	Arg 48
**B7a**	18S	664	28S	1693-1695, 1725
**B7b/c**	18S	737-738, 675-677	uL2 (RPL8)	Lys 155, Arg 149, Gly 248
**B8**	18S	322-324	uL14 (RPL23)	Asn 31
**eB8**	absent			
**eB11**	eS8	Glu 99, Arg 102	28S	1594-1595, 2651-2652
**eB12**	18S	602-605, 609	eL19	Arg 173, Val 177, Lys 171
**eB13**	eS6	Asp 37	eL24	Asp 67
**eB14**	18S	1425-1432, 858-860, 867-868, 1327-1328	eL41	Lys 21, Arg 17, Arg 11, Arg 9, Lys 7, Lys 4, Arg 2

*Contacts within 4 Å radius were selected using the program Contact (34, 35) and were verified by visual inspection of the refined structure.
